# Repeated exposure to methamphetamine induces sex-dependent hypersensitivity to ischemic injury in the adult rat heart

**DOI:** 10.1371/journal.pone.0179129

**Published:** 2017-06-02

**Authors:** Boyd R. Rorabaugh, Sarah L. Seeley, Thorne S. Stoops, Manoranjan S. D’Souza

**Affiliations:** Department of Pharmaceutical and Biomedical Sciences, College of Pharmacy, Ohio Northern University, Ada, Ohio, United States of America; University of Cincinnati College of Medicine, UNITED STATES

## Abstract

**Background:**

We previously reported that adult female, but not male rats that were prenatally exposed to methamphetamine exhibit myocardial hypersensitivity to ischemic injury. However, it is unknown whether hypersensitivity to ischemic injury develops when rats are exposed to methamphetamine during adulthood. The goal of this study was to determine whether methamphetamine exposure during adulthood sensitizes the heart to ischemic injury.

**Methods:**

Adult male and female rats received daily injections of methamphetamine (5 mg/kg) or saline for 10 days. Their hearts were isolated on day 11 and subjected to a 20 min ischemic insult on a Langendorff isolated heart apparatus. Cardiac contractile function was measured by an intraventricular balloon, and infarct size was measured by triphenyltetrazolium chloride staining.

**Results:**

Hearts from methamphetamine-treated females exhibited significantly larger infarcts and suppressed postischemic recovery of contractile function compared to hearts from saline-treated females. In contrast, methamphetamine had no effect on infarct size or contractile recovery in male hearts. Subsequent experiments demonstrated that hypersensitivity to ischemic injury persisted in female hearts following a 1 month period of abstinence from methamphetamine. Myocardial protein kinase C-ε expression, Akt phosphorylation, and ERK phosphorylation were unaffected by adult exposure to methamphetamine.

**Conclusions:**

Exposure of adult rats to methamphetamine sex-dependently increases the extent of myocardial injury following an ischemic insult. These data suggest that women who have a heart attack might be at risk of more extensive myocardial injury if they have a recent history of methamphetamine abuse.

## Introduction

Methamphetamine is one of the most commonly abused illicit stimulants in the United States. According to the 2015 National Survey on Drug Use and Health, 6% of survey respondents over 26 years of age reported using methamphetamine at some point during their life time [1]. Methamphetamine abuse is associated with an increased risk of adverse cardiovascular events including stroke [2, 3], cardiac arrhythmias [2, 4], subarachnoid and intracerebral hemorrhage [3], and methamphetamine-induced cardiomyopathy [5, 6]. Methamphetamine can also induce myocardial ischemia leading to a heart attack [7–10]. However, the impact of methamphetamine on the ischemic heart has not been extensively studied.

We recently reported that prenatal exposure to methamphetamine induces sex-dependent effects in the heart that persist into adulthood [11]. Hearts from adult female rats that had been prenatally exposed to methamphetamine exhibited significantly larger infarcts following an ischemic insult compared to hearts from adult females that were prenatally exposed to saline [11]. This was accompanied by changes in proteins (PKC-ε expression and Akt phosphorylation) that are known to protect the heart from ischemic injury [11]. In contrast, prenatal methamphetamine had no effects on infarct size, PKC-ε expression, or Akt phosphorylation in their adult male littermates [11]. These data provide evidence that prenatal exposure to methamphetamine leads to sex-dependent changes in cardioprotective proteins and increased myocardial sensitivity to ischemic injury that persists into adulthood. However, it is unknown whether the myocardial hypersensitivity to ischemic injury that is observed in adult hearts that were prenatally exposed to methamphetamine also occurs when rats are exposed to methamphetamine as adults. Thus, the goal of this study was to determine whether methamphetamine induces myocardial hypersensitivity to ischemic injury when rats are exposed to the drug as adults.

## Methods

### Animals

Sprague Dawley rats used for this study were obtained from a breeding colony maintained at Ohio Northern University. The rat colony originated from Sprague Dawley rats obtained from Charles River Laboratories (Boston, MA) Strain Code 001. Rats were pair housed in standard Plexiglas cages with free access to food and water and were maintained on a 12 hour / 12 hour light / dark schedule (lights on at 07:00). All procedures were approved by the Institutional Animal Care and Use Committee of Ohio Northern University.

### Subchronic treatment of adult rats with methamphetamine

Naïve adult male and female rats (8 weeks of age) were subcutaneously injected once daily (at 10:00) with methamphetamine (5 mg/ kg/day) or saline for 10 consecutive days. This dose was used based on our previous work demonstrating that methamphetamine treatment of pregnant female rats produces myocardial changes in their adult offspring [11]. This dose is also commonly used by other investigators to study the effects of methamphetamine-induced neurological and behavioral effects [12–14], and it is within the range of methamphetamine doses (on a mg/kg basis) typically used by people in illicit settings [15]. The subchronic duration (10 days) of this treatment was based on a recent study demonstrating that repeated exposure of rats to methamphetamine over the course of 10 days worsens cerebral injury in a rat model of ischemic stroke [16]. Hearts were isolated on day 11 (24 hours after the last methamphetamine or saline injection), mounted on a Langendorff isolated heart apparatus, and subjected to an ischemic insult as described below. For some experiments, hearts were isolated 1 month after the last methamphetamine injection to investigate the effects of a 1 month period of abstinence.

### Treatment of adult rats with a single exposure to methamphetamine

Naïve adult female rats (8 weeks of age) were subcutaneously injected once (at 10:00) with methamphetamine (5 mg / kg) or saline. Their hearts were isolated 24 hours later, mounted on a Langendorff isolated heart apparatus, and subjected to an ischemic insult as described below.

### Langendorff isolated heart experiments

Rats were anesthetized with sodium pentobarbital (100 mg/kg ip), and their hearts were rapidly removed and mounted on a Langendorff isolated heart apparatus as previously described [11]. Krebs solution (in mM: 118 NaCl, 4.7 KCl, 1.2 MgSO_4_, 25 NaHCO_3_, 1.2 KH_2_PO_4_, 0.5 Na_2_EDTA, 11 glucose, and 2.5 CaCl_2_, pH 7.4) was perfused through an aortic cannula at a constant pressure of 80 mmHg. Contractile function of the left ventricle was measured using an intraventricular balloon connected to a pressure transducer. The balloon was inflated to an end-diastolic pressure of 4 mmHg, and data were continuously recorded using a Powerlab 4SP data acquisition system (AD Instruments, Colorado Springs, CO). The heart was submerged in Krebs solution to maintain the temperature of the heart at 37.5 ± 0.5°C throughout the experiment, and temperature was continuously monitored using a thermocouple placed on the surface of the heart. Hearts were equilibrated for 25 min prior to the onset of 20 min of ischemia and 2 hours of reperfusion. Hearts were excluded after the 25 minute equilibration period if developed pressure was <100 mmHg, coronary flow rate was >25 ml/min, or if there were persistent arrhythmias. Preischemic contractile function was measured immediately prior to ischemia, and postischemic recovery of contractile function was measured following 1 hour of reperfusion. Hearts were perfused for an additional hour (2 hours of total reperfusion) prior to triphenyltetrazolium chloride staining.

### Measurement of infarct size

Hearts were perfused with 1% triphenyltetrazolium chloride at a rate of 7.5 ml/min for 8 min and then submerged in 1% triphenyltetrazolium chloride at 37°C for 12 min. Hearts were subsequently frozen at −80°C, sliced into 1-mm sections, soaked in 10% neutral buffered formalin, and then photographed with a Nikon SMZ 800 microscope equipped with a Nikon DS-Fi1 digital camera. The infarcted surface area and total surface area of each slice were measured using ImageJ software (National Institutes of Health). Infarct size was expressed as a percentage of the entire ventricular myocardium.

### Western blotting

Basal levels of PKC-ε expression were measured on day 11 following 10 days of treatment with saline or methamphetamine (5 mg/kg) injections. Rats were anesthetized with sodium pentobarbital (75 mg/kg ip), and hearts were quickly isolated and perfused for 5 minutes on a Langendorff isolated heart system to flush blood from the tissue. The left ventricle was immediately flash frozen in liquid nitrogen and stored at −80°C until it was used for western blotting with antibodies for PKC-ε (Santa Cruz Biotechnology, Dallas, TX; catalog # sc-214) and glyceraldehyde-3-phosphate dehydrogenase (GAPDH) (Cell Signaling Technology, Danvers, MA; catalog # 2118). PKC-ε bands were normalized to GAPDH as previously described [11].

Akt and ERK phosphorylation were measured in serial ventricular biopsies taken from perfused hearts immediately prior to ischemia, following 20 minutes of ischemia, and following 5 minutes of reperfusion. Ventricular biopsies were flash frozen in liquid nitrogen and stored at −80°C until they were used for western blotting as previously described [11].

### Statistical analysis

Infarct sizes were compared by two way ANOVA and Bonferoni’s posthoc analysis using sex (male vs female) and drug treatment (methamphetamine vs saline) as factors. For experiments in which parameters of cardiac function (developed pressure, +dP/dT, -dp/dT, heart rate, and end diastolic pressure, and coronary flow rate) were measured in both male and female rats, data were analyzed by three-way ANOVA and Bonferonni’s posthoc analysis with time (preischemic vs postischemic recovery analyzed as repeated measures), sex (male vs female), and drug treatment (methamphetamine vs saline) as factors. PKC-ε expression was analyzed by the Student’s t test (saline vs methamphetamine treatment). Akt and ERK phosphorylation were analyzed by two way-ANOVA and Bonferonni’s posthoc test with time (repeated measure before ischemia, during ischemia, and during reperfusion) and drug treatment (methamphetamine vs saline) as factors.

## Results

### Repeated exposure of adult rats to methamphetamine produces sex-dependent effects on infarct size and postischemic recovery of contractile function

**Fig 1:**
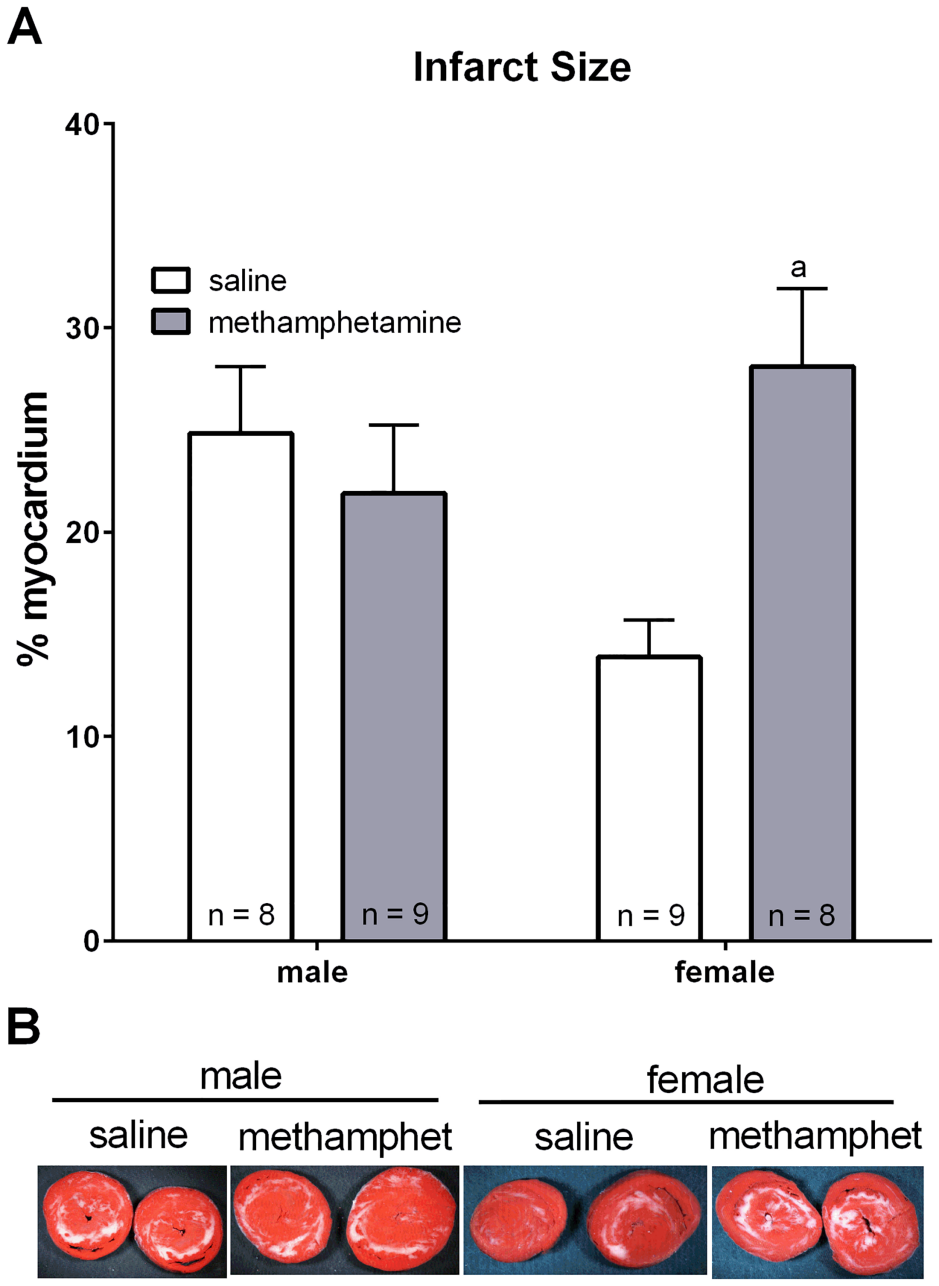
Infarct sizes in male and female hearts from rats treated for 10 days with methamphetamine or saline. Rats received subcutaneous injections of saline or methamphetamine (5 mg/kg/day) for 10 consecutive days. Hearts were isolated 24 hours after the final injection and subjected to 20 minutes ischemia and 2 hours of reperfusion on a Langendorff heart apparatus. Hearts were stained with triphenyltetrazolium chloride, and infarct sizes were measured as described in the Methods. (**A**) Two way ANOVA identified a significant interaction [F = 7.6 (1, 30), p < 0.01] between sex (male vs female) and methamphetamine treatment (saline vs methamphetamine). ^a^ indicates a significant difference (p < 0.05) compared to saline-treated females. Panel **B** show representative photographs of heart slices from each experimental group. Data represent the mean ± S.E.M. of 8–9 hearts.

The impact of methamphetamine on the ischemic heart was measured following 10 days of methamphetamine exposure. On day 11, hearts were subjected to 20 minutes ischemia followed by reperfusion. Two-way ANOVA indicated that treatment of adult rats with methamphetamine differentially impacted infarct sizes in male and female hearts [significant interaction between sex and methamphetamine exposure; F = 7.6 (1, 30), p < 0.01]. Hearts from methamphetamine-treated females had significantly larger infarcts than hearts from saline-treated females (Fig 1). In contrast, methamphetamine had no effect on infarct size in male hearts (Fig 1). Infarcts in hearts from saline-treated female rats were nominally smaller than those of saline-treated males, but this did not reach statistical significance.

**Fig 2:**
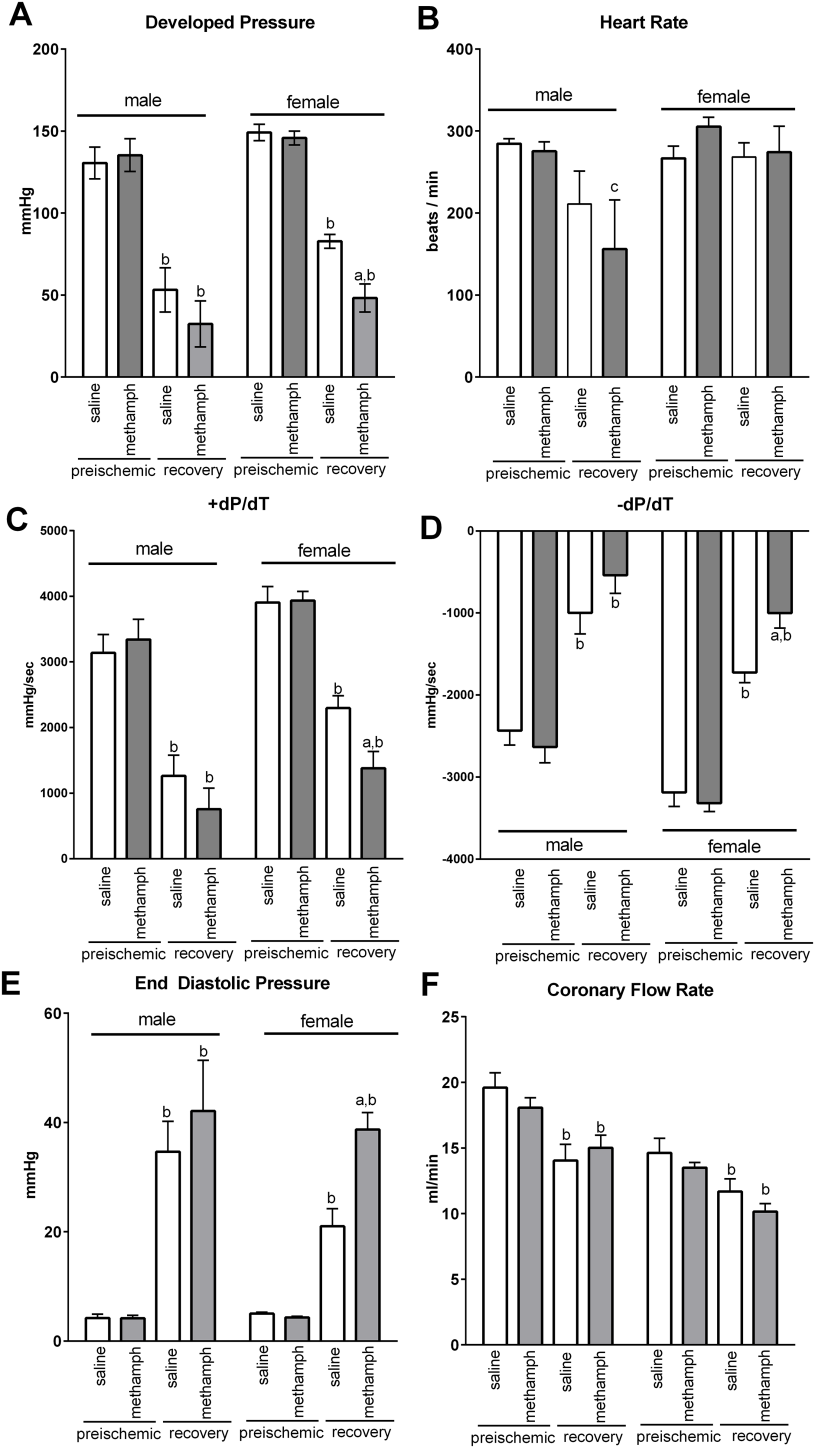
Parameters of preischemic contractile function and postischemic recovery of contractile function in male and female hearts treated for 10 days with either methamphetamine or saline. Preischemic contractile function was measured following a 25 minutes equilibration period immediately prior to the onset of ischemia. Postischemic recovery was measured following 1 hour of reperfusion. Three way ANOVA indicated a significant effect of time (preischemic vs postischemic recovery) for developed pressure [F = 193 (1, 34), p < 0.001] (**A**), heart rate [F = 7.5 (1, 34), p < 0.01] (**B**), +dP/dT [F = 167 (1, 34) p < 0.001] (**C**), -dP/dT [F = 250 (1, 34) p < 0.001] (**D**), end diastolic pressure [F = 111 (1, 34), p < 0.001] (**E**), and coronary flow rate [F = 82 (1, 34), p < 0.001] (**F**). There was also a significant interaction between time and methamphetamine treatment for developed pressure [F = 5.4 (1, 34), p < 0.05], +dP/dT [F = 6.0 (1, 34), p = 0.02], -dP/dT [F = 7.7 (1, 34), p < 0.01], and end diastolic pressure [F = 5.5 (1, 34), p < 0.05]. There was a significant effect of sex [F = 34 (1, 34), p < 0.001] on coronary flow rate. Bars represent the mean ± S.E.M of hearts from 8–10 rats. ^a^ indicates a significant difference (p < 0.05) compared to postischemic recovery of hearts from saline-treated females. ^b^ indicates a significant difference (p < 0.001) compared to the preischemic value. ^c^ indicates a significant difference (p < 0.05) compared to the preischemic value.

Ten days of methamphetamine treatment had no effect on preischemic parameters of contractile function including developed pressure (Fig 2A), heart rate (Fig 2B), +dP/dT (Fig 2C), -dP/dT (Fig 2D), or end diastolic pressure (EDP) (Fig 2E) in hearts isolated from either male or female rats. However, methamphetamine differentially influenced postischemic recovery of contractile function in male and female hearts. Postischemic recovery of developed pressure (Fig 2A), +dP/dT (Fig 2C), and -dP/dT (Fig 2D) were significantly suppressed in hearts isolated from methamphetamine-treated female rats compared to hearts from saline-treated females. Furthermore, postischemic EDP was significantly elevated in female hearts treated with methamphetamine compared to hearts from saline-treated females (Fig 2E), indicating that they were unable to relax appropriately during diastole. In contrast, postischemic recovery of contractile function was unaffected by methamphetamine in male hearts. The finding that methamphetamine worsens postischemic recovery of both systolic (Fig 2A and 2C) and diastolic (Fig 2D and 2E) contractile function in female hearts (but not in male hearts) is consistent with the observation that infarct size (Fig 1) was increased in female hearts (but not male hearts) that had been repeatedly exposed to methamphetamine.

Methamphetamine had no effect on heart size. As expected, male hearts (saline-treated male hearts = 1,221 ± 37 mg; methamphetamine-treated male hearts = 1,193 ± 47 mg) were larger than female hearts (saline-treated female hearts = 847 ± 91 mg; methamphetamine-treated female hearts = 884 ± 24 mg). However, methamphetamine had no significant impact on heart weight in either sex.

### A single exposure to methamphetamine has no effect on infarct size or postischemic recovery of contractile function

In order to determine whether myocardial hypersensitivity to ischemic injury can be induced by a single exposure to methamphetamine, female rats were given a single subcutaneous injection of either methamphetamine (5 mg/kg) or saline 24 hours prior to inducing myocardial ischemia in isolated hearts. Infarct sizes in hearts from saline- treated rats (12 ± 3% of myocardium, n = 8) and methamphetamine-treated rats (13 ± 4% of myocardium, n = 8) were similar. A single exposure to methamphetamine also had no significant effect on preischemic or postischemic recovery of contractile function (Table 1). These data indicate that repeated exposure to methamphetamine is necessary to induce myocardial hypersensitivity to ischemic injury. The impact of a single exposure to methamphetamine was not examined in male hearts because male hearts were unaffected by repeated exposure to this drug (Figs 1 and 2).

**Table 1:** Parameters of preischemic and postischemic recovery of contractile function in female hearts 24 hours after a single exposure to saline or methamphetamine.

	Before Ischemia		Postischemic Recovery	
	Saline	Methamphetamine	Saline	Methamphetamine
**Developed Pressure (mmHg)**	136 ± 10	144 ± 12	82 ± 7^b^	91 ± 16^b^
**Heart Rate (bpm)**	261 ± 24	312 ± 11	234 ± 25	266 ± 38
**+dP/dT (+mmHg/sec)**	3995 ± 436	4561 ± 523	2433 ± 321^b^	2760 ± 562^c^
**-dP/dT (-mmHg/sec)**	-2841 ± 307	-3248 ± 259	-1743 ± 170^a^	-1921 ± 328^b^
**End diastolic Pressure (mmHg)**	5 ± 1	4 ± 1	27 ± 3^b^	28 ± 5^c^
**Coronary Flow (ml/minute)**	12 ± 1	13 ± 1	9 ± 1	10 ± 0.4

Cardiac contractile function was measured 24 hours following treatment with a single injection of either saline or methamphetamine (5 mg/kg). Data were analyzed by repeated measures two way ANOVA with treatment (saline vs methamphetamine) and time (repeated measure before ischemia vs postischemic recovery) as factors. Data represent the mean ± S.E.M of 8 hearts for each group. There were significant effects of time on developed pressure [F = 55 (1, 14), p < 0.0001], +dP/dT [F = 59 (1, 14), p < 0.0001], -dP/dT [F = 49 (1, 14), p < 0.0001], end diastolic pressure [F = 71 (1, 14), p < 0.0001], and coronary flow rate [F = 11 (1, 14), p < 0.005].

^a^ indicates a significant difference (p < 0.01) compared to the preischemic value.

^b^ indicates a significant difference (p < 0.005) compared to the preischemic value.

^c^ indicates a significant difference (p < 0.0001) compared to the preischemic value.

### Methamphetamine-induced myocardial hypersensitivity to ischemic injury persists in female hearts following one month of abstinence

**Fig 3:**
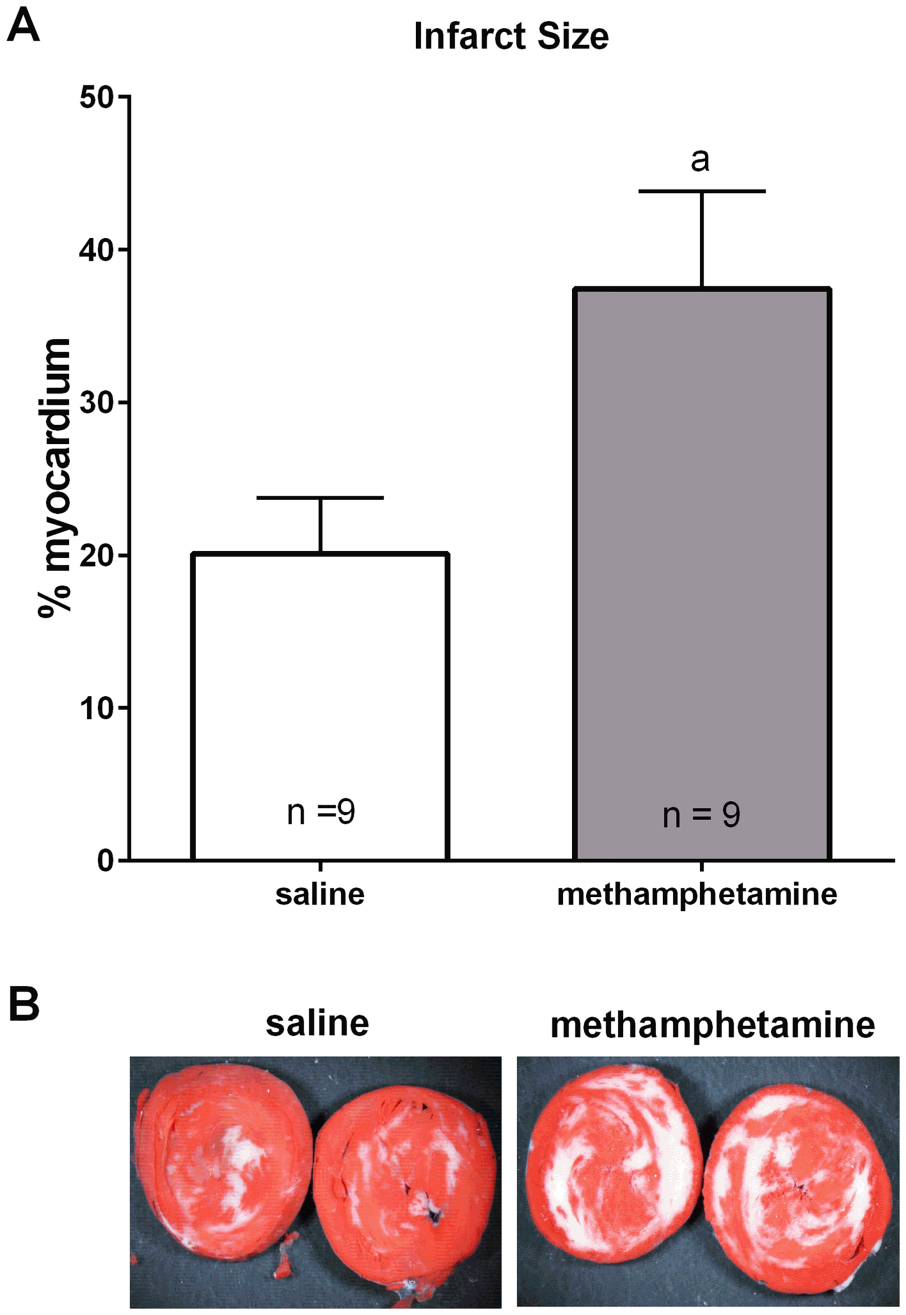
Infarct sizes in female hearts following 1 month of abstinence from methamphetamine. Female rats received daily subcutaneous injections of methamphetamine (5 mg/kg) or saline for 10 consecutive days. Heart were isolated following 1 month of abstinence from saline or methamphetamine and subjected to 20 minutes ischemia and 2 hours reperfusion on a Langendorff isolated heart apparatus. (**A**) Hearts were stained with triphenyltetrazolium chloride, and infarct sizes were measured as described in the Methods. Infarct sizes were analyzed by the student’s t test. ^a^ indicates p < 0.05. Data represent the mean ± S.E.M. of 9 hearts. Panel **B** show representative photographs of heart slices from each experimental group.

We also examined the impact of methamphetamine on the ischemic heart following a 1 month period of abstinence from the drug. Adult (8 weeks of age) female rats received daily subcutaneous injections of saline or methamphetamine (5 mg/kg) for 10 consecutive days followed by 1 month of abstinence. Infarct sizes were significantly larger in hearts from methamphetamine-treated females compared to saline-treated females (Fig 3). Postischemic recovery of contractile function was similar in methamphetamine and saline-treated hearts following 1 month of abstinence (Table 2). The persistence of methamphetamine’s effect was not measured in males because male hearts were not affected by 10 days of methamphetamine exposure (Figs 1 and 2).

**Table 2:** Parameters of preischemic and postischemic recovery of contractile function in female hearts following 10 days of saline or methamphetamine exposure followed by 1 month of abstinence.

	Before Ischemia		Postischemic Recovery	
	Saline	Methamphetamine	Saline	Methamphetamine
**Developed Pressure (mmHg)**	127 ± 5	132 ± 9	70 ± 11^b^	58 ± 8^c^
**Heart Rate (bpm)**	262 ± 8	274 ± 14	229 ± 32	263 ± 15
**+dP/dT (+mmHg/sec)**	3693 ± 269	4086 ± 352	2067 ± 399^b^	1747 ± 203^c^
**-dP/dT (-mmHg/sec)**	-2954 ± 210	-3074 ± 195	-1327 ± 252^c^	-1230 ± 142^c^
**End diastolic Pressure (mmHg)**	5 ± 0.6	5 ± 0.3	27 ± 5^a^	31 ± 5^b^
**Coronary Flow (ml/minute)**	14 ± 0.6	13 ± 0.8	11 ± 0.7^a^	11 ± 0.5^a^

Rats received daily injections of saline or methamphetamine (5 mg/kg) for 10 days. Cardiac contractile function was measured following 1 month of abstinence from saline or methamphetamine. Data were analyzed by repeated measures two way ANOVA with treatment (saline vs methamphetamine) and time (repeated measure before ischemia vs postischemic recovery) as factors. Data represent the mean ± S.E.M of 9 hearts for each group. There were significant effects of time on developed pressure [F = 59 (1, 16), p < 0.0001], +dP/dT [F = 59 (1, 16), p < 0.0001], -dP/dT [F = 74 (1, 16), p < 0.0001], end diastolic pressure [F = 40 (1, 14), p < 0.0001], and coronary flow rate [F = 18 (1, 16), p < 0.005].

^a^ indicates a significant difference (p < 0.01) compared to the preischemic value.

^b^ indicates a significant difference (p < 0.005) compared to the preischemic value.

^c^ indicates a significant difference (p < 0.0001) compared to the preischemic value.

### Repeated exposure of adult female rats to methamphetamine has no effect on myocardial PKC-ε expression

**Fig 4:**
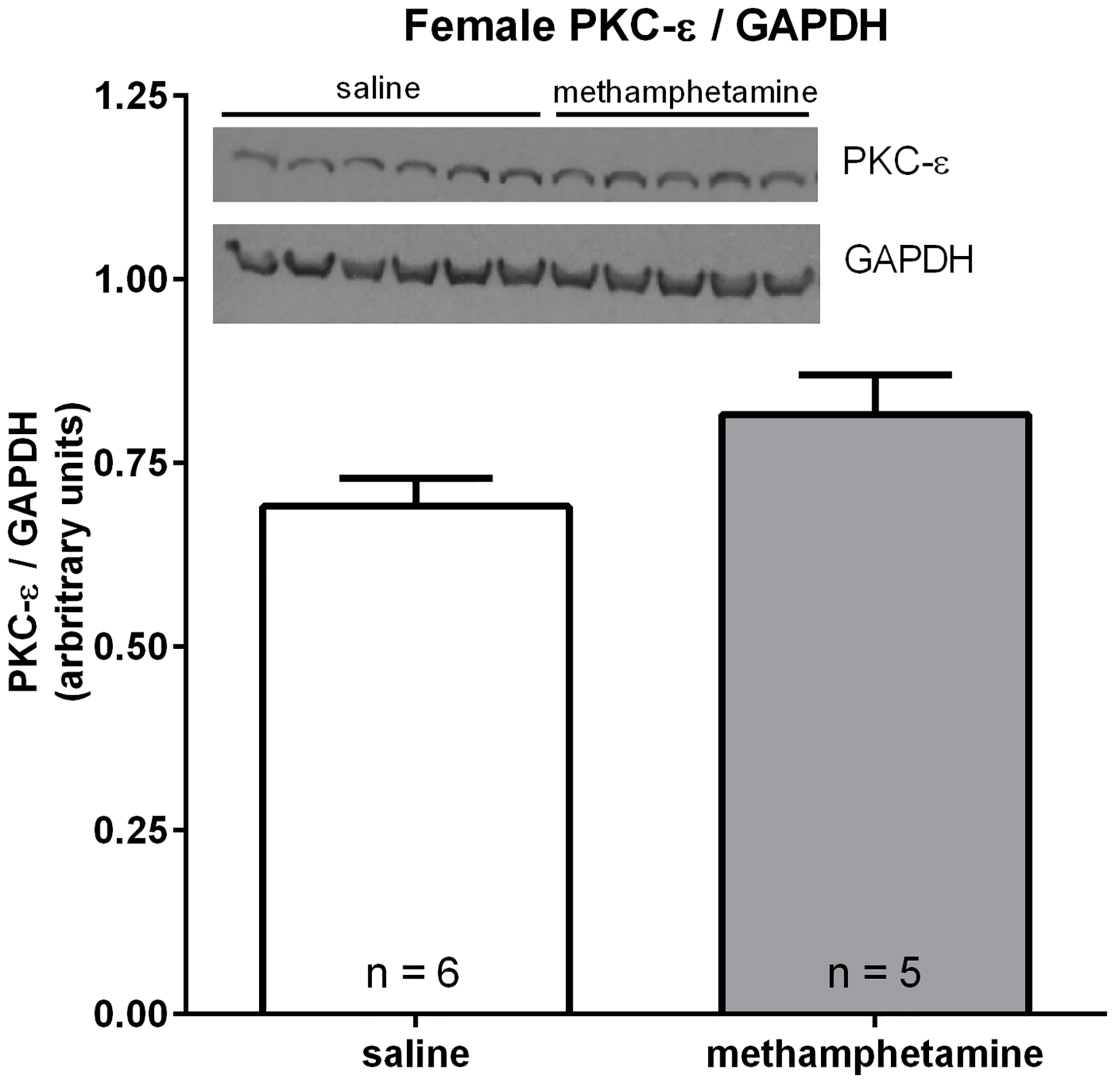
Subchronic methamphetamine has no effect on PKC-ε expression in the ventricular myocardium of female rats. Female rats received daily subcutaneous injections of methamphetamine (5 mg/kg) or saline for 10 consecutive days. Hearts were isolated on day 11 and immediately mounted on a Langendorff isolated heart apparatus. Blood was flushed from the coronary vasculature by perfusing the hearts with Krebs solution for 5 minutes. The tissue was subsequently flash frozen in liquid nitrogen and analyzed by western blotting. Band intensities were quantified by NIH Image J software. PKC-ε expression was normalized to glyceraldehyde-3-phosphate dehydrogenase. Bars represent the mean ± S.E.M. of hearts from 5–6 different rats. Data were analyzed by the student’s t test.

We previously reported that adult female rats (but not males) that were prenatally exposed to methamphetamine exhibit suppressed myocardial PKC-ε expression and decreased phosphorylation of Akt [11]. Thus, we assessed the impact of adult exposure to methamphetamine on the basal expression of this cardioprotective protein. Rats received daily subcutaneous injections of methamphetamine (5 mg / kg) or saline for 10 days and hearts were isolated for western blotting on day 11. Unlike prenatal exposure to methamphetamine [11], exposure to methamphetamine during adulthood had no impact on myocardial PKC-ε expression (Fig 4).

### Methamphetamine has no impact on ERK or Akt phosphorylation in the ischemic heart

**Fig 5:**
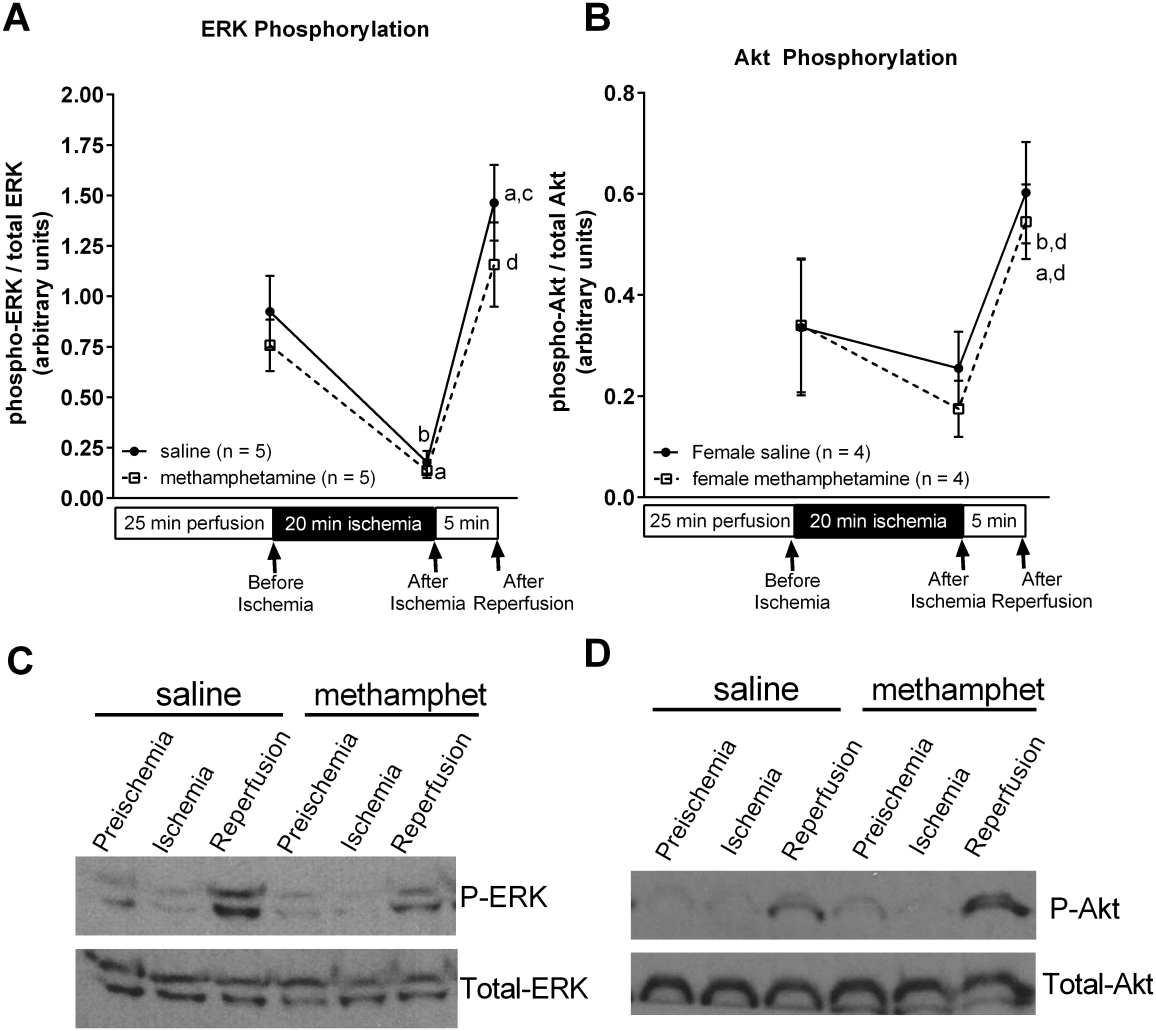
Methamphetamine has no effect on ERK or Akt phosphorylation in the ventricular myocardium before ischemia, during ischemia, or following 5 minutes reperfusion. Female rats received daily subcutaneous injections of methamphetamine (5 mg/kg) or saline for 10 consecutive days. Hearts were isolated on day 11 and perfused on a Langendorff isolated heart apparatus. Serial ventricular biopsies were isolated from each heart and flash frozen in liquid nitrogen following 25 minutes perfusion, following 20 minutes ischemia, and after 5 minutes reperfusion. Western blots were used to measure phospho-ERK, total ERK (**A**), phospho-Akt and total Akt (**B**). Band intensities were quantified by NIH Image J software. Phospho-ERK band intensities were normalized to that of total ERK band intensities (**A**), and phospho-Akt band intensities were normalized to those of total Akt (**B**). Representative blots of phospho-ERK and total ERK are shown in panel **C**. Representative blots of phospho-Akt and total Akt are shown in panel **D**. Data were analyzed by two-way repeated measures ANOVA with treatment (saline vs methamphetamine) and time (repeated measure) as factors. There were significant overall effects of time on both ERK phosphorylation [F = 32 (2, 16), p < 0.0001] (**A**) and Akt phosphorylation [F = 31 (2, 12), p < 0.0001] (**B**). ^a^ indicates a significant difference (p < 0.05) compared to phosphorylation before ischemia. ^b^ indicates a significant difference (p < 0.01) compared to phosphorylation before ischemia. ^c^ indicates a significant difference (p < 0.001) compared to phosphorylation after ischemia. ^d^ indicates a significant difference (p < 0.005) compared to phosphorylation after ischemia.

Akt and ERK have well established roles as cardioprotective proteins [17–19]. Phosphorylation of these proteins at the time of reperfusion triggers endogenous signaling mechanisms that protect the heart from reperfusion-induced injury [19]. Thus, we investigated the possibility that methamphetamine may worsen myocardial injury by interfering with Akt or ERK signaling. Following 10 days of treatment with saline or methamphetamine, ERK and Akt phosphorylation were measured (on day 11) in serial biopsies obtained from the left ventricle prior to the onset of ischemia, following 20 minutes ischemia, and following 5 minutes reperfusion. There was a significant effect of time on both ERK [F = 32 (2, 16), p < 0.0001] and Akt [F = 31 (2, 12), p < 0.0001] phosphorylation. ERK phosphorylation was significantly decreased following 20 minutes ischemia compared to its preischemic value (Fig 5A) in hearts from both saline and methamphetamine-treated animals. This was followed by a significant increase in ERK phosphorylation following 5 min reperfusion. There was also a reperfusion-induced increase in Akt phosphorylation in hearts from both saline and methamphetamine-treated rats (Fig 5B). However, reperfusion-induced increases in ERK and Akt phosphorylation were unaffected by repeated methamphetamine exposure. These data indicate that methamphetamine-induced hypersensitivity to ischemic injury is not the result of interference with reperfusion-induced phosphorylation of these cardioprotective proteins.

## Discussion

We recently reported that hearts from adult female rats that were prenatally exposed to methamphetamine are hypersensitive to ischemic injury [11]. Surprisingly, their adult male littermates were unaffected by prenatal methamphetamine exposure. The present study extends this previous work by demonstrating that methamphetamine exposure during early adulthood also sensitizes the heart to ischemic injury and that this occurs in a sex-dependent manner. Other investigators have also reported that females are more sensitive than males to some effects of methamphetamine. Methamphetamine produces a greater increase in locomotor activity in female rats and mice than in males [20–21]. In addition, female rats that have been previously exposed to methamphetamine are more likely than males to self-administer methamphetamine when they have free access to the drug [22]. Females are also more likely than males to display drug seeking behavior after a period of forced abstinence from methamphetamine [23]. However, this is the first study to demonstrate that adult exposure to methamphetamine produces a sex-dependent effect in the heart.

Prenatal exposure to methamphetamine leads to decreased PKC-ε expression and myocardial hypersensitivity to ischemic injury in adult female rats [11]. In contrast to prenatal exposure, female rats that were directly exposed to methamphetamine during adulthood exhibited myocardial hypersensitivity to ischemic injury without diminished PKC-ε expression (Fig 4), suggesting that prenatal and adult exposure to methamphetamine sensitize the female heart to ischemic injury through different mechanisms. It is unclear why PKC-ε expression is suppressed in adult female hearts following prenatal exposure to methamphetamine but not in hearts that were directly exposed to methamphetamine during adulthood. The developing fetus is sensitive to stimuli that induce epigenetic changes in DNA, resulting in changes in gene expression that persist into adulthood [24]. Prior work has demonstrated that PKC-ε expression is suppressed in hearts of adult rats that had been prenatally exposed to hypoxia [25–26], nicotine [27], cocaine [28], or methamphetamine [11]. Prenatal exposure to most of these stimuli is known to suppress PKC-ε expression by inducing methylation of nucleotides in the promoter region of the gene that encodes PKC-ε, resulting in decreased transcription of the PKC-ε gene [25–26, 29–30]. In contrast to prenatal exposure, we are unaware of any studies demonstrating that exposure of rats to these stimuli during adulthood suppresses PKC-ε expression. Thus, the observation that prenatal exposure, but not adult exposure, to methamphetamine suppresses PKC-ε expression might reflect the greater sensitivity of the prenatal heart to epigenetic changes in gene expression.

Previous studies from our own lab and the work of others indicate that female hearts are less susceptible than male hearts to ischemic injury [11, 31–33]. This has been attributed to sex differences in the activity of ATP-dependent potassium channels [33], the mitochondrial permeability transition pore [34], and differences in nitric oxide-dependent cardioprotective signaling pathways [35]. In addition, estrogen is known to protect the heart from ischemic injury [36–37]. Shen et al. [38] recently reported that function of the hypothalamic-pituitary-ovarian axis is disrupted in women who chronically use methamphetamine and that it remains disrupted following several months of abstinence from the drug. Thus, methamphetamine might negate the cardioprotective benefit of being female by disrupting the hypothalamic-pituitary-ovarian axis and suppressing the production of cardioprotective ovarian hormones.

The behavioral effects of methamphetamine primarily result from methamphetamine-induced efflux of catecholamines through the dopamine transporter (DAT) and norepinephrine transporter (NET) which are expressed in dopaminergic and sympathetic neurons. Females have greater DAT expression than males in some regions of the brain including the striatum [39–40] and the substantia nigra [39]. Females also exhibit higher rates of striatal dopamine release and dopamine reuptake through DAT than males [41]. These sex differences in the molecular target of methamphetamine are likely to be responsible for some of the sex-dependent effects of methamphetamine that are mediated through the central nervous system. However, methamphetamine also produces direct effects in the heart that might be responsible for enhancing myocardial ischemic injury. Mitochondria play a critical role in ischemic injury [42]. Recent studies indicate that methamphetamine increases oxidative stress in both cultured rat cardiac myocytes and in isolated mitochondria, leading to the oxidation of membrane lipids and the nitration of proteins [43–44]. These events result in the opening of the mitochondrial permeability transition pore which triggers the activation of proapoptotic proteins and lead to cell death [43–46]. These direct effects of methamphetamine on the heart may underlie the myocardial hypersensitivity to ischemic injury that was observed in our study. Additionally, our finding that methamphetamine selectively targets female hearts could simply be the result of sex differences in the pharmacokinetics of methamphetamine. Clearance of methamphetamine occurs at a slower rate in adult female rats compared to adult males [47]. This results in a greater area under the concentration-time curve for females (compared to males), resulting in females experiencing more exposure to methamphetamine than males given the same dose of the drug [47]. This increased exposure to methamphetamine in females may be the cause of the sex-dependent effect of methamphetamine in the ischemic heart. In summary, the sex-dependent effect of methamphetamine on the ischemic heart could be the result of sex differences in the expression or function of proteins (ATP-dependent potassium channels, mitochondrial permeability transition pore, nitric oxide synthase, etc.) that modulate myocardial sensitivity to ischemic injury [33–35], methamphetamine-induced changes in the release of cardioprotective female sex hormones, or sex differences in the pharmacokinetics of methamphetamine that cause females to have greater cardiac exposure to methamphetamine. The observation that hypersensitivity to ischemic injury persists following a month of abstinence suggests that the mechanism involves changes in gene expression or other processes that are not rapidly reversible. Further work is needed to identify the mechanism by which methamphetamine selectively worsens ischemic injury in the female heart.

Methamphetamine has been previously shown to produce behavioral and neurological effects (impaired memory, decreased social interaction, decreased firing of neurons in the prefrontal cortex, changes in the expression of synaptosomal proteins) in rodents that persist following a prolonged period of abstinence from the drug [48–51]. The present data demonstrate for the first time that treatment of adult rats with methamphetamine also produces cardiac effects that persist following an extended period (1 month) of abstinence. This is consistent with our previous finding that female rats which were prenatally exposed to methamphetamine exhibit myocardial hypersensitivity to ischemic injury following 2 months of postnatal abstinence [11]. We have not examined the duration of this effect beyond 1 month (following exposure of adult rats) or 2 months (following prenatal exposure). Thus, it is unknown whether the myocardial impact of methamphetamine exposure that occurs during the prenatal [11] or early adult (present study) period persists into the geriatric phase of life when a heart attack is most likely to occur. This is a limitation of this study.

Induction of myocardial ischemia immediately following 10 days of methamphetamine treatment (no period of abstinence) increased infarct size (Fig 1) and significantly worsened postischemic recovery of contractile function (Fig 2). Hearts from female rats subjected to 10 days methamphetamine and a one month period of abstinence prior to myocardial ischemia also displayed increased infarct size relative to hearts from saline-treated controls rats (Fig 3). These hearts also exhibited nominally worsened postischemic recovery of contractile function for most parameters of contractile function (Table 2), but these differences did not reach statistical significance. Consistent with this outcome, other investigators have also reported that increased infarct size is not always accompanied by significant worsening of contractile recovery [52–54]. This has been attributed to myocardial stunning in which tissue that is not dead (stains red with triphenyltetrazolium chloride) does not contract properly [52]. Furthermore, decreased postischemic flow rates that are generally observed during reperfusion can mask the true contractile function of the myocardium. For these reasons, infarct size is generally regarded as more reliable than contractile function as an indicator of cardiac injury [52–54].

The impact of methamphetamine on ischemic injury is not limited to the heart. Zuloaga et al. [16] recently reported that chronic methamphetamine treatment increases the extent of cerebral injury in a mouse model of ischemic stroke. Mice injected with methamphetamine for 10 consecutive days prior to transient occlusion of the middle cerebral artery demonstrated significantly larger cerebral infarcts and worsened cognitive function compared to control mice that were injected with saline. These effects were observed in male mice (females were not studied) which differs from our work in the ischemic heart where we observed an effect of methamphetamine only in females. The observation that methamphetamine worsens ischemic injury in the male brain but not the male heart might result from the fact that methamphetamine concentrations are significantly higher in the brain than in peripheral tissues following subcutaneous injection [55].

Our data raise important concerns regarding the impact of methamphetamine use on the ischemic heart. It is difficult to determine whether methamphetamine induces a state of hypersensitivity to ischemic injury in the human heart because methamphetamine use in humans is frequently accompanied by other cardiovascular risk factors such as poor diet and the use of tobacco [56]. Unlike ischemic insults induced in the laboratory, human infarcts differ with respect to their duration, anatomical location within the heart, and the amount of myocardial tissue that is affected by the coronary blockage. Thus, the heterogenous nature of ischemic insults in the clinical setting contributes to the difficulty in determining whether methamphetamine increases the extent of ischemic injury in the human heart. However, our data suggest that women who experience a heart attack might be at risk of more extensive myocardial injury if they have a history of recent methamphetamine use (present study) or if they were prenatally exposed [11] to methamphetamine.
